# A multi-client web platform for telemetry of the mechanical properties of material

**DOI:** 10.1016/j.ohx.2026.e00741

**Published:** 2026-01-10

**Authors:** Oleg Ivanov, Volodymyr Shulgin, Nataliia Popovych, Liudmyla Bondar

**Affiliations:** Poltava State Agrarian University, Skovorody Street 1/3, Poltava 36003, Ukraine

**Keywords:** Wireless web server, Web application, Websocket, Telemetry, Linear potentiometer, Mechanical properties of materials

## Abstract

This paper presents a multi-client web platform for telemetry of mechanical property testing, built around an existing tensile testing machine as an approach to its modernization. Extension and force measurements are performed using potentiometers with analog outputs connected to a 24-bit analog-to-digital converter (ADS1256) integrated with a Raspberry Pi 5 microcomputer. The communication module enables data acquisition and timing, the organization of a wireless Wi-Fi network, the operation of web and DNS servers, and full-duplex information exchange via the WebSocket protocol.

A specially developed web application with a graphical user interface allows researchers to observe the “force–extension” curve and numerical values in real time on personal digital devices without installing specialized software. The platform supports simultaneous multi-client access, which increases the accessibility and convenience of experimental research.

Validation experiments on reinforcing steel confirmed stable platform performance at a data acquisition frequency of 15 Hz with up to 15 simultaneous client connections. The measurement accuracy was within ± 1 % for both force and extension, meeting ISO 6892–1 requirements. The proposed solution provides an affordable and flexible tool for scientific and educational purposes, enhancing the informativeness and accuracy of experimental tests while offering a practical pathway for upgrading conventional tensile testing machines.

## Graphical abstract

**Figure:**
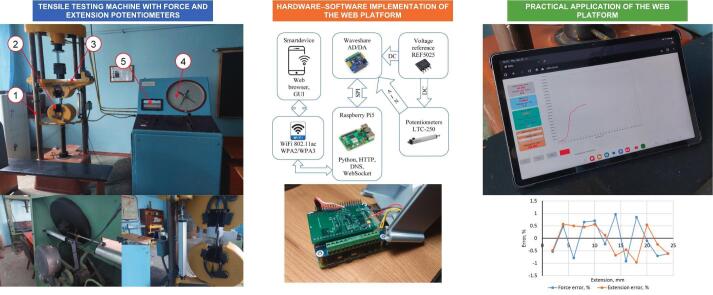

Specifications table

**Table:** 

Hardware name	*A Multi-client Web Platform for Telemetry of the Mechanical Properties of Material*
Subject area	*Engineering and materials science*
Hardware type	*Measuring physical properties and in-lab sensors*
Closest commercial analog	No commercial analog is available
Open source license	Creative Commons Attribution-ShareAlike 4.0 International License (CC BY-4.0)
Cost of hardware	$397
Source file repository	https://doi.org/10.17605/OSF.IO/MPN2Y

## 1 Hardware in context

The determination of the mechanical properties of materials is of paramount importance for modern science and engineering, as these properties define the suitability of a material for specific structural and technological applications. The reliability and durability of machines, structures, and products directly depend on accurate knowledge of strength characteristics such as yield strength, tensile strength, and elastic modulus, which serve as the basis for engineering calculations and modeling of loading conditions. Experimental testing using both contact and non-contact methods enables the assessment of material quality, the detection of hidden defects, and the assurance of operational safety [1]. Such investigations are particularly important for novel and composite materials [2], [3], where experimental methods often remain the only means of confirming their compliance with international standards [4]. The obtained results form the foundation for standardization, certification, and industrial implementation of materials, as well as provide the scientific basis for the development of mathematical models of extension and fracture.

Given the importance of obtaining reliable strength characteristics, the instrumentation employed for such investigations is of particular significance. The technical configuration of testing systems determines the accuracy, reproducibility, and informational value of the results. At present, the key testing systems for determining the mechanical properties of materials are universal testing machines [5] equipped with electromechanical, mechanical, or hydraulic loading actuators. Such equipment enables the application of controlled forces and the registration of extension parameters; however, traditional data recording systems based on mechanical chart recorders and paper media exhibit significant limitations in terms of accuracy, response speed, and subsequent data processing.

The rapid advancement of digital technologies, microcontroller systems, and sensor devices open new opportunities for modernizing testing equipment. One of the key directions of such modernization is the replacement or supplementation of mechanical measurement systems with precision extension and force sensors, which provide high accuracy and stable readings over a wide measurement range [6]. For force measurement, strain gauge sensors [7] are most commonly used, as they are characterized by high sensitivity and relatively low cost. Pneumatic and hydraulic pressure sensors are also employed, particularly in systems designed for high-force applications. For dynamic processes, the use of piezoelectric sensors [8] is advisable, whereas magnetoelastic sensors [9] provide reliability under demanding operational conditions. To determine extension, linear potentiometric sensors, inductive and capacitive transducers, strain-gauge extensometers, as well as video extensometers based on machine vision technologies [10] are applied. Each of these sensor types has its own advantages and limitations, which define their suitability for specific experimental conditions.

Equally important is the organization of data acquisition and visualization. Traditional approaches with local parameter recording significantly limit the possibilities for real-time analysis and collaborative data use. Modern testing systems should provide automated acquisition of measurement data with high temporal resolution, its storage in digital format, and accessibility for analysis by a broad community of users.

Commercial tensile testing systems such as Instron Bluehill, Zwick/Roell TestXpert, Tinius Olsen Horizon, and data-acquisition platforms based on National Instruments hardware offer high-precision measurement subsystems with certified sensors, high sampling rates, advanced closed-loop control, and comprehensive analytical software. However, these systems are expensive, proprietary, and difficult to integrate with legacy mechanical machines. Modernizing older pendulum-based or mechanical tensile machines typically requires full replacement of the measurement subsystem, which represents a substantial financial burden and limits accessibility for small laboratories and educational institutions.

A different approach is presented in [11], where a cloud-based remote monitoring and control system is developed for a high-temperature fatigue testing machine. The platform relies on Browser/Server architecture and uses a public cloud server providing data storage, WebSocket communication, and video streaming. Although this system achieves low network latency and high automation levels, it requires continuous access to external cloud infrastructure, incurs maintenance costs, and depends on global internet connectivity. Furthermore, the solution is tailored to a specific class of equipment and is not intended for low-cost retrofitting of existing mechanical testing machines.

Additional insight into low-cost and open hardware solutions can be found in recent developments based on Raspberry Pi microcomputers. In particular, the system described in [12] proposes an autonomous Raspberry Pi–based spectrometer that integrates a camera module, optical waveguide, and spectroscope into a compact measurement device. The authors developed a Python application using Tkinter, OpenCV, and PIL libraries to perform real-time acquisition, visualization, and digital post-processing of spectral data. Their calibration methodology is based on direct comparison with a reference spectrometer (Black Comet C-SR-50), supplemented by laser and mercury lamp spectra, and employs polynomial error-correction functions to improve wavelength reconstruction accuracy. This work demonstrates the potential of low-cost embedded platforms to replace commercial instrumentation while achieving acceptable metrological performance for scientific, educational, and environmental monitoring applications.

In contrast to both cloud-based remote testing architectures [11] and specialized embedded measurement devices such as the Raspberry Pi spectrometer [12], the platform proposed in this study is designed as a locally deployed, fully autonomous, browser-based telemetry system specifically tailored for retrofitting legacy tensile testing machines.

This combination of local autonomy, low implementation cost, open hardware/software architecture, and compatibility with existing mechanical equipment distinguishes the proposed platform from commercial DAQ systems, cloud-based remote monitoring solutions, and other Raspberry-Pi-based scientific instruments reported in the literature. It therefore fills an important technological niche: modernization of legacy tensile machines with minimal financial and infrastructural requirements while providing a flexible and scalable measurement environment suitable for laboratory teaching and research.

The use of smartphones, tablets, and laptops equipped with built-in wireless modules allows direct access to measurement data during experiments. This eliminates the need for specialized terminals and simplifies research organization, as almost every researcher already owns a device that can display data in a web browser. Another advantage is the ability to connect multiple devices at the same time. This facilitates collective observation of test results in real time. Overall, this approach increases accessibility and convenience and meets modern requirements for mobility, interactivity, and scalability in scientific research.

In this context, there is a clear need to develop an information and communication system that integrates measurement sensors, microcontroller-based signal digitization, and wireless data exchange technologies to acquire, transmit, and visualize data in real time.

The aim of this study is to design a distributed web platform for telemetry of the mechanical properties of materials using personal digital devices. This platform will improve the informativeness and accuracy of experiments while providing convenient access to data for both researchers and students.

To achieve this aim, the following tasks must be addressed:

‒ To equip the tensile testing machine with precision sensors for recording extension and applied force, as well as audiovisual tools for documenting the testing process;

‒ To develop a digital module for signal digitization and data acquisition based on a microcontroller with Wi-Fi support and wireless data transmission capabilities;

‒ To design a web application for managing the testing process, monitoring parameters in real time, and visualizing the obtained results;

‒ To implement distributed data access to allow multiple users to use the system simultaneously.

## 2 Hardware description

The described distributed web platform improves the efficiency and convenience of experiments on the mechanical properties of materials. It enables real-time remote monitoring of applied force and extension parameters. It also provides visualization and offers simultaneous access to experimental data for multiple users.

The web platform consists of:

‒ Sensor and measurement equipment for tracking changes in experimental parameters;
‒ A communication module with an integrated ADC, digitizing and transmitting data via a wireless network;
‒ A software shell with a graphical user interface (GUI), enabling control, data processing, and real-time visualization

A detailed presentation and description of each platform component are provided below.

### 2.1 Sensor and measurement equipment

**Fig. 1:**
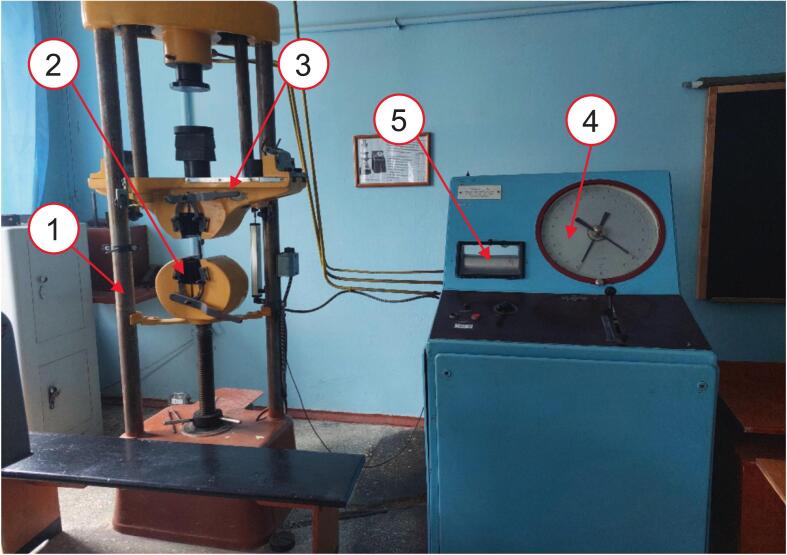
Tensile testing machine.

The developed web platform represents an integrated solution for an existing tensile testing machine. The tensile testing machine (Fig. 1) is used for static testing of metallic and other specimens under tension, compression, bending, and tight bending. It consists of a vertical two-column frame, a lower fixed mechanical grip (2), a vertically movable crosshead (3), a pendulum force gauge (4), and a mechanical chart recorder (5).

When testing specimens under tension with this machine, two parameters are monitored: the extension of the specimen clamped between the lower grip and the crosshead, and the applied force on the specimen. A mechanical chart recorder translates these parameters into the position of a coordinate point on paper. The collection of these points forms the characteristic tensile curve of the specimen. Based on this curve, the mechanical properties of the material are subsequently evaluated using a graphical–analytical method.

When selecting sensors, it is essential that they provide high measurement accuracy across the full range of these parameters. The tensile machine can apply forces from 0 to 200 kN, while the absolute measurement error must not exceed 100 N according to the international testing standard [13]. The extension can vary within 0–100 mm, with a measurement accuracy of 0.01 mm. Both parameters are physically represented as the linear displacement of specific moving elements. The instantaneous coordinate of each element determines the corresponding parameter value. Linear potentiometers were therefore selected because they directly convert this displacement into a proportional voltage.

**Fig. 2:**
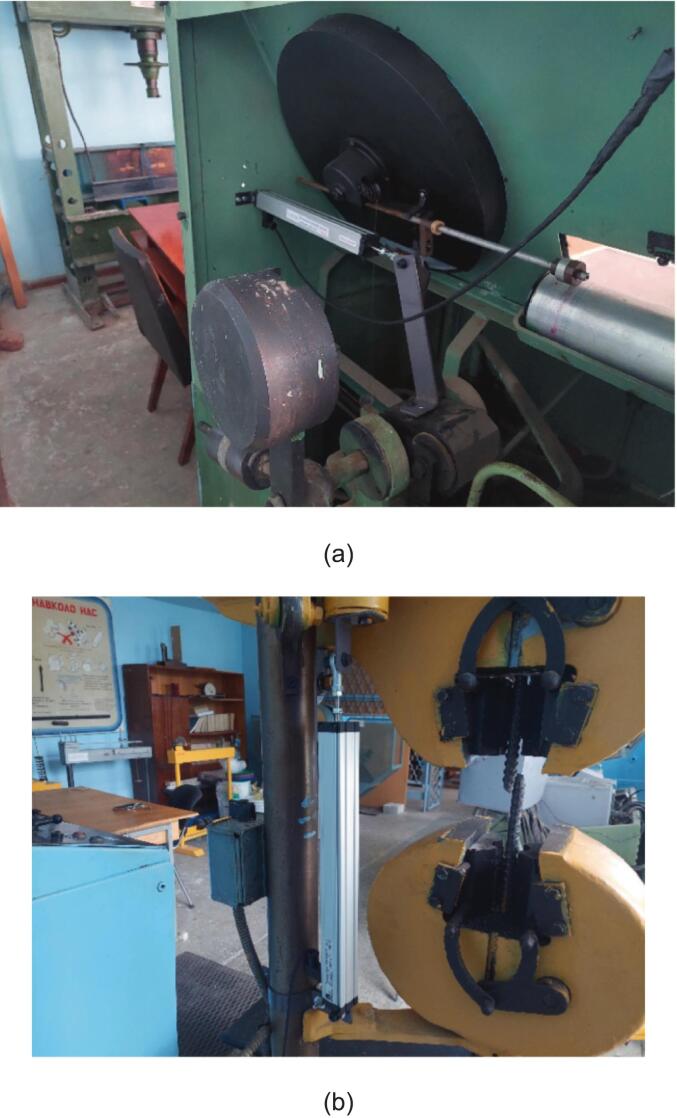
Placement of potentiometers in the tensile testing machine: (a) potentiometer for force measurement; (b) potentiometer for extension measurement.

To monitor these parameters, two LTC-250 linear potentiometers were employed. One potentiometer measures the displacement of the pendulum force transducer (Fig. 2a), whose position at any given moment corresponds to the applied force. The other potentiometer (Fig. 2b) tracks the displacement of the crosshead relative to the fixed frame, thereby characterizing the extension of the specimen. This potentiometer is mounted between the lower fixed mechanical grip and the moving crosshead. To ensure galvanic isolation, the potentiometers are attached to the metallic base of the tensile testing machine using plastic fastening elements fabricated on a 3D printer. Such isolation separates the electrical potentials of the machine and the sensors, prevents stray currents between components, and ensures stable system operation.

The output voltage level of the potentiometers depends on the positions of the connected crosshead and pendulum force transducer. It determines the values of the specimen’s extension and applied force.

To ensure the reliability of the measurement subsystem, both linear potentiometers underwent a full calibration procedure in a certified metrological laboratory compliant with ISO 10012:2003, “Measurement management systems – Requirements for measurement processes and measuring equipment.” All calibration procedures were conducted under controlled environmental conditions: an ambient temperature of (20 ± 1) °C and relative humidity below 60 %, in accordance with standard metrological practice.

Calibration of the extension measurement channel was carried out according to ISO 13385–1:2019, “Geometrical product specifications (GPS) – Dimensional measuring equipment.” A set of micrometers with different measurement ranges and a resolution of 0.01 mm was used as reference instruments. The calibration methodology involved recording the potentiometer’s output voltage as a function of the linear displacement of the sensor rod. The rod displacement was controlled by a micrometer head rigidly fixed to the potentiometer rod. This ensured that the actual displacement values were traceable to the calibrated micrometers. The resulting voltage–displacement dataset was used to construct an approximation equation describing the relationship between the measured output voltage and the actual extension of the specimen.

Calibration of the force measurement channel followed ISO 7500–1:2018, “Metallic materials – Calibration and verification of static uniaxial testing machines.” A reference force transducer (dynamometer) of accuracy class 0.5 served as the standard. During calibration, the output voltage of the potentiometer attached to the pendulum force transducer was recorded at multiple force levels. These force levels were applied and verified using the reference dynamometer. The resulting voltage–force pairs were processed to obtain an approximation equation accurately representing the functional dependence between the sensor output and the applied force.

For both measurement channels, correlation analysis was performed to evaluate linearity, determine regression parameters, and quantify residual deviations. The obtained calibration curves were used to convert raw voltage values into extension and force during all subsequent experiments. The calibration results confirmed that the combined uncertainty introduced by sensor nonlinearity, reference instrument tolerances, ADC conversion, and environmental factors remained within the limits required for laboratory-grade tensile testing.

The approximated dependence for extension:

(1) e=-0.667857·U+251.808

where e – extension, mm;

U – voltage, mV.

Approximate dependence for applied force:

(2) $$F=2\cdot 10^{-13}\cdot U^{2}-704.75\cdot U+239727$$

where F – applied force, N.

This approach allows indirect determination of the applied force on the test specimen and the extension it induces.

The calibration curves presented in Eqs. (1), (2) were obtained from dedicated calibration experiments conducted separately for the extension and force measurement channels. For each channel, a series of controlled force or displacement steps was applied. The corresponding potentiometer output voltage was recorded. In both cases, 10 calibration points were collected uniformly across the full operating range of the testing machine.

For the extension channel, a linear regression model was applied. This is because the LTC-250 potentiometer used for displacement sensing exhibits a highly linear characteristic. The resulting approximation yielded a slope of –0.0667857 mV/mm and an intercept of 251.808 mV. The coefficient of determination was R^2^ = 0.9987. Analysis of the residuals showed no systematic trends, confirming the suitability of a linear model.

For the force channel, the mechanical structure of the pendulum force transducer introduces measurable geometric nonlinearity. Therefore, a second-order polynomial regression was selected as the most appropriate model. The calibration dataset produced a quadratic fit with R^2^ = 0.9973. The residual distribution remained within ± 0.8 % of the full-scale output without observable bias.

These regression models form the basis of Eqs. (1), (2). They enable accurate indirect determination of the applied force and induced extension from the measured potentiometer voltage.

### 2.2 Communication module with integrated ADC

The web platform is built on a typical client–server architecture and consists of several levels:

‒ Hardware level – responsible for high-precision digitization of signals from sensors and time-stamping measurements;
‒ Network level – based on a Wi-Fi 802.11ac wireless network, providing connectivity with users’ personal devices. Data transmission security is ensured by WPA2/WPA3 protocols using a secret access key. This level also blocks requests to external servers, preventing unauthorized data transfer and protecting against cyberattacks;
‒ Server level – responsible for web server operation. It handles requests via HTTP/HTTPS and transmits hypertext data (HTML, CSS, JS). A DNS server enables access via a local domain name. The WebSocket protocol establishes a full-duplex, real-time communication channel for data exchange;
‒ Client level – provides a web application with a graphical user interface (GUI) in users’ browsers. It supports multi-client operation and compatibility with standard browsers.

The first three levels are implemented within the communication module.

**Fig. 3:**
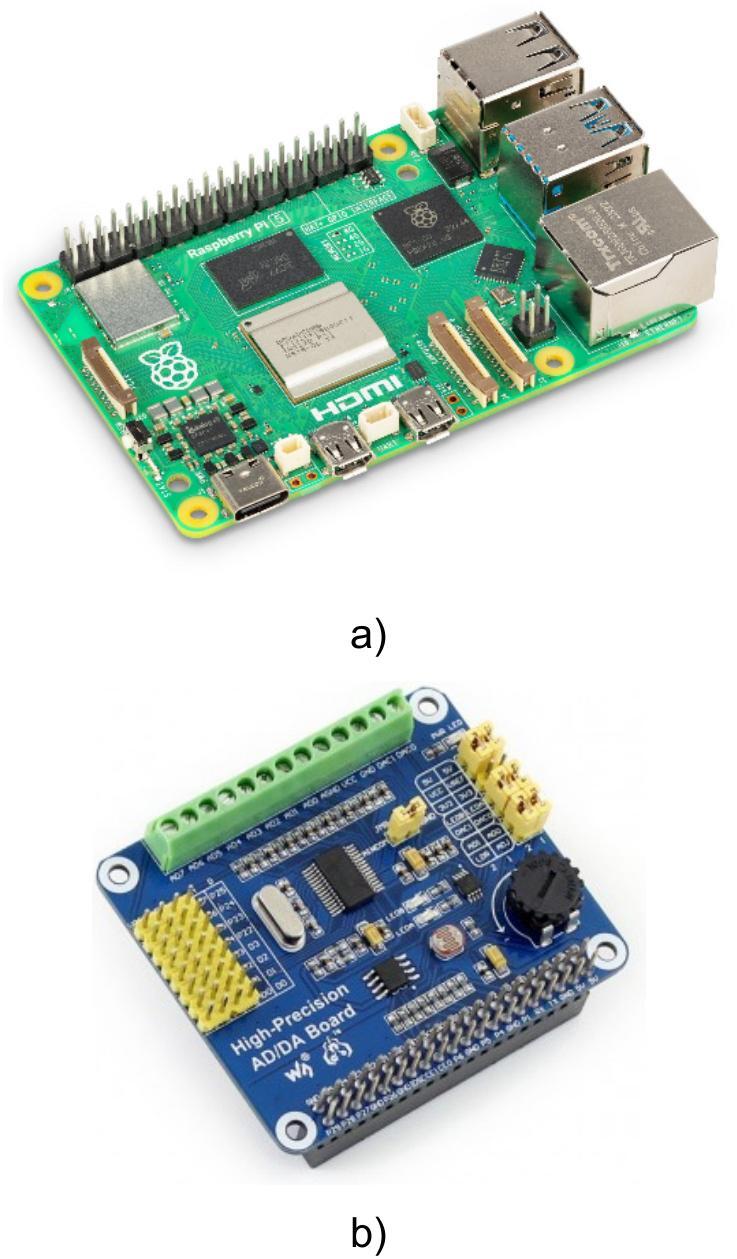
Components of the communication module: (a) Raspberry Pi 5 microcomputer; (b) Waveshare AD/DA module with ADS1256 ADC.

The module is based on a Raspberry Pi 5 microcomputer with 4 GB of RAM (Fig. 3a). It is powered by a quad-core ARM Cortex-A76 processor with a 2.4 GHz clock frequency. To convert analog signals from the potentiometers, an external Waveshare AD/DA module (Fig. 3b) was employed. This module features a 24-bit ADC ADS1256 by Texas Instruments. The ADC supports up to 8 analog inputs (4 differential), allowing scalability and the connection of additional sensors.

The ADC reference voltage source was an external precision voltage reference REF5025 with a 2.5 V output. It was mounted on a prototyping board together with passive components (e.g., filtering capacitors). The ADC was connected to the voltage reference via wired conductors, while communication with the Raspberry Pi 5 was established through an SPI bus. This ensures high-speed data exchange and reliable software integration. A built-in Wi-Fi 802.11ac module provides stable networking and distributed access to measurement data.

The Wi-Fi network, together with the web server, enables the use of standard HTTP/HTTPS protocols. It also supports the WebSocket protocol, which is compatible with most personal devices and browsers. This setup eliminates the need for additional communication interfaces or specialized software on users’ devices.

### 2.3 Graphical user interface.

**Fig. 4:**
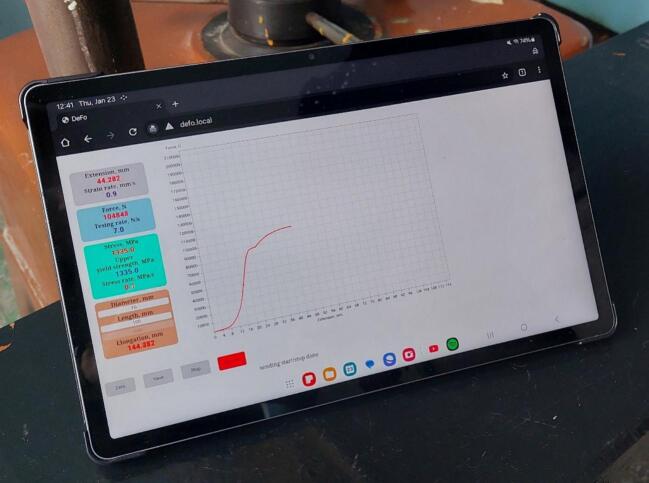
Display of the web page in the browser of a personal digital device.

For practical implementation of the described architecture, a dedicated web application was developed to enable user interaction with the platform. The server side of the web application operates on the Raspberry Pi 5 communication module. The client side is implemented as a web page in the browser of a personal digital device (Fig. 4).

The HTML code of the web page, together with the required scripts and style sheets, is loaded from the web server of the communication module via an HTTP request. The request uses a local domain name (e.g., “defo.local”) assigned by the DNS server. This eliminates the need to memorize an IP address and makes the system convenient to use.

The graphical user interface (GUI) has a structured layout that combines text and graphical blocks. Text blocks are grouped by parameters and visually distinguished by color to improve readability. They display real-time numerical values of technical parameters and their rates of change. For example, blocks show paired values such as extension–strain rate, force–testing rate, and stress–stress rate. Interactive < input > elements allow the user to enter key geometrical characteristics of the specimen, such as cross-sectional area and gauge length. These values are processed by JavaScript as constants for subsequent strength calculations.

The graphical block of the web page is based on the < canvas > element, where the force–extension curve is plotted via JavaScript. In the coordinate system, extension (mm) is plotted along the x-axis and applied force (N) along the y-axis. This arrangement allows researchers to monitor the testing process in real time and correlate numerical values with their graphical representation.

The GUI also includes interactive control buttons for managing the testing process. Specifically:

‒ Zero – resets sensor readings before the test.
‒ Save – stores experimental data in digital format.
‒ Start/Stop – initiates or terminates the measurement process.

These control elements make the testing process flexible and user-friendly. They enable researchers to fully manage experiments directly from their personal digital devices.

The dataset acquired during testing can be saved locally in a CSV file with tab-delimited values. The graphical representation of the force–extension curve can be stored as a PNG raster image. These files can be used in various software environments for further analysis.

The developed GUI ensures intuitive interaction with the platform. It combines numerical monitoring, real-time visualization, and convenient data management. The GUI provides flexibility, multi-client accessibility, and integration with standard web technologies. These features together enhance the usability of the experimental setup.

## 3 Design files summary

**Table:** 

**Design file name**	**File type**	**Open source license**	**Location of the file**
Plastic fastening elements for potentiometers	*CAD files*	*CC BY-4.0*	*(Folder: 3D fasteners)* https://doi.org/10.17605/OSF.IO/MPN2Y
Plastic enclosure for the Raspberry Pi 5 microcomputer and the analog-to-digital converter module	*CAD files*	CC BY-4.0	*(Folder: 3D* case for an electronic device*)* https://doi.org/10.17605/OSF.IO/MPN2Y
Python code for the Raspberry Pi 5 microcomputer	Python program files	CC BY-4.0	*(Folder:* Software Raspberry Pi5 (python software)) https://doi.org/10.17605/OSF.IO/MPN2Y
HTML and CSS code of the web page with the graphical user interface	HTML, CSS code	CC BY-4.0	*(Folder:* Software GUI (HTML&CSS&JS)) https://doi.org/10.17605/OSF.IO/MPN2Y
Raspberry Pi operating system image	Software	CC BY-4.0	*(Folder: ImageOS)* https://doi.org/10.17605/OSF.IO/MPN2Y

Plastic fastening elements for potentiometers are 3D-printed components. They ensure the secure mounting of potentiometers in the tensile testing machine and provide galvanic isolation between the sensors and the machine.

A plastic enclosure for the Raspberry Pi 5 microcomputer and the ADC module is a 3D-printed housing. It protects these electronic components from mechanical damage and integrates them into a single unit.

Python code for the Raspberry Pi 5 microcomputer consists of a set of scripts for managing the communication module. These scripts include the ADS1256 driver via SPI, a data acquisition and time-stamping loop, the implementation of WebSocket/HTTP/DNS servers, and client command handling.

HTML and CSS code of the web page with the graphical user interface are essential for running the web page on the user’s personal digital device. They include HTML code for structure, embedded CSS styles for design, and JavaScript scripts for interactivity and server communication.

The Raspberry Pi operating system image is a Raspberry Pi OS image with preinstalled Python, a WebSocket server, required libraries (RPi.GPIO, spidev), and mDNS configuration.

## 4 Bill of materials summary

**Table:** 

**Designator**	**Component**	**Number**	**Cost per unit −currency**	**Total cost −** **currency**	**Source of materials**	**Material type**
Microcomputer	Raspberry Pi5	1	$120	$120	https://www.raspberrypi.com/products/raspberry-pi-5/	Electronics
Analog-to-digital conversion module for potentiometer signals	Waveshare AD/DA	1	$35	$35	https://www.waveshare.com/wiki/High-Precision_AD/DA_Board	Electronics
Precision voltage reference	REF5025	1	$8	$8	https://www.ti.com/product/REF5025-HT?keyMatch = ref5025&tisearch = universal_search#product-details	Electronics
Potentiometer	LTC-250	2	$110	$220	https://ateksensor.com/en/kategori/linear-measurement/potentiometric-linear-position-sensors/	Metal and Electronics
Set of plastic fastening elements for potentiometers	bolts, nuts, washers	4	$1	$4	3D-printed part	Polylactic acid (PLA) (3D-printed material)
Enclosure for the microcomputer and analog-to-digital conversion module	Case	1	$10	$10	3D-printed part	Polylactic acid (PLA) (3D-printed material)

### 4.1 Build instructions

The deployment process of the developed multi-client web platform involves several steps. These include mounting potentiometers on the tensile testing machine, assembling the hardware components of the platform, and installing and configuring the software on the Raspberry Pi 5 microcomputer.

The extension potentiometer is fixed to the lower stationary mechanical grip using plastic fastening elements. The potentiometer rod is attached to the movable crosshead with plastic fastening elements (Fig. 2b).

**Fig. 5:**
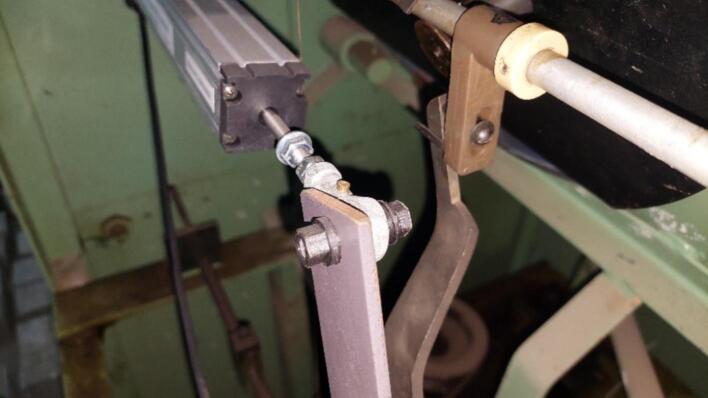
Plastic fastening elements integrated with potentiometer mounting part.

The force potentiometer is mounted within the mechanism of the pendulum force transducer. Its housing is connected to the solid metal body of the force transducer, and the rod is attached to its lever (Fig. 2a). Plastic fastening elements also ensure galvanic isolation from the mechanical parts of the tensile testing machine (Fig. 5).

The ADC module is connected to the Raspberry Pi 5 microcomputer via a 40-pin connector. This connector is inserted into the corresponding socket on the board.

The contact pads of the ADC module are connected with colored wires grouped into a 4-pin external connector. The combined potentiometer plug is attached to this connector. The red wire supplies the positive terminal (Vcc) of the precision power source. The black wire carries the negative terminal (GND). The yellow wire serves as the input for the first ADC channel, and the white wire serves as the input for the second channel.

**Fig. 6:**
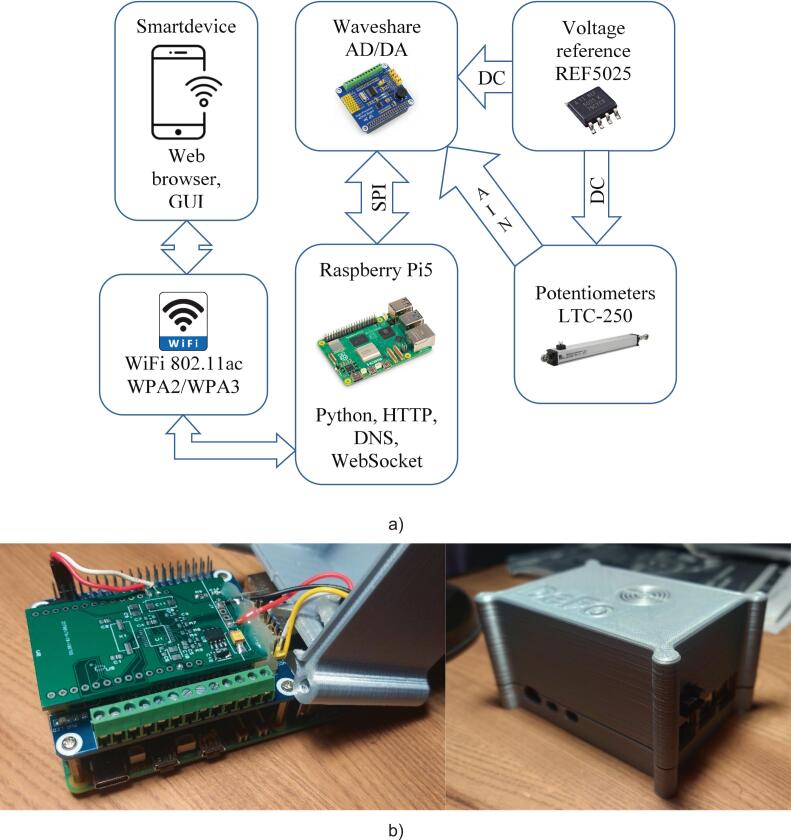
Communication module: (a) architecture; (b) physical layout.

The microcomputer, ADC module, and 4-pin connector are placed in a 3D-printed plastic enclosure. The general architecture of the communication module is shown in Fig. 6a, and its physical layout is shown in Fig. 6b.

Each potentiometer has its own electrical connector on the housing and a plug for cable attachment. Each plug is connected to a shielded three-wire cable. Two conductors provide power supply (Vcc and GND), and one serves as the output signal line. Since both potentiometer connectors have the same pinout and require a precision power source, the cables are routed and soldered into a single shared connector. This wiring arrangement allows the potentiometers and the ADC module to automatically share a common ground (GND). This ensures measurement accuracy and prevents parasitic potential differences.

**Fig. 7:**
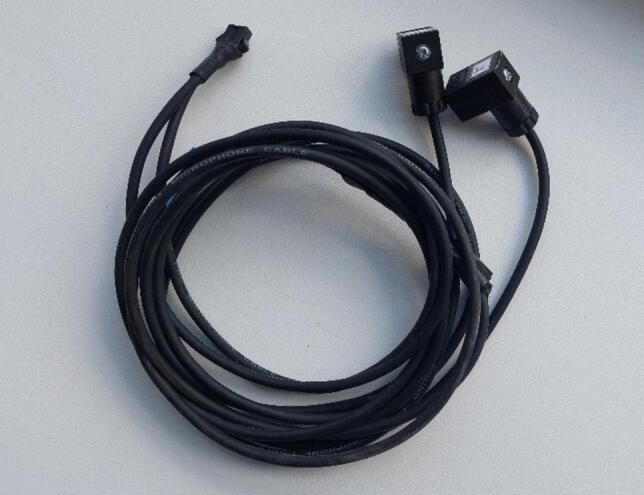
Potentiometer cable.

The assembled cable with connectors is shown in Fig. 7.

The next stage of deploying the web platform is installing and configuring the software on the Raspberry Pi 5 microcomputer. For rapid setup on a new Raspberry Pi 5, a preconfigured operating system image with preinstalled software components is used. Installation is performed by writing this image onto a microSD card using Raspberry Pi Imager or similar utilities.

After inserting the microSD card into the Raspberry Pi 5 slot and supplying power, the system automatically boots with the preinstalled environment. This environment includes drivers for the ADC module, server components (web server, DNS server, WebSocket module), and Python scripts for data acquisition and processing.

This approach provides a fully operational environment. It reduces setup time and eliminates the need for manual software installation. It also minimizes the risk of installation errors and ensures reproducibility of experiments across different laboratory conditions.

If necessary, updates to the Python code or the source web code of the graphical user interface can be performed using standard file replacement methods on the microcomputer via the terminal. The latest code versions are provided in the sources listed in the table of Section 3.

## 5 Operation instructions

The methodology for applying the developed web platform in experimental studies of the strength characteristics of materials using a tensile testing machine is as follows.

The test specimen is clamped between the lower mechanical grip and the crosshead of the machine. The potentiometers are connected to the hardware part of the communication module via a connector. Then, the module is powered from a DC power source. Such a source may be a battery-based device that ensures a stable, low-noise power supply and autonomous operation.

After power is supplied, a Wi-Fi network appears in the 2.4 GHz band. Researchers connect to this network using an access code. This allows them to join the platform from their personal digital devices.

**Fig. 8:**
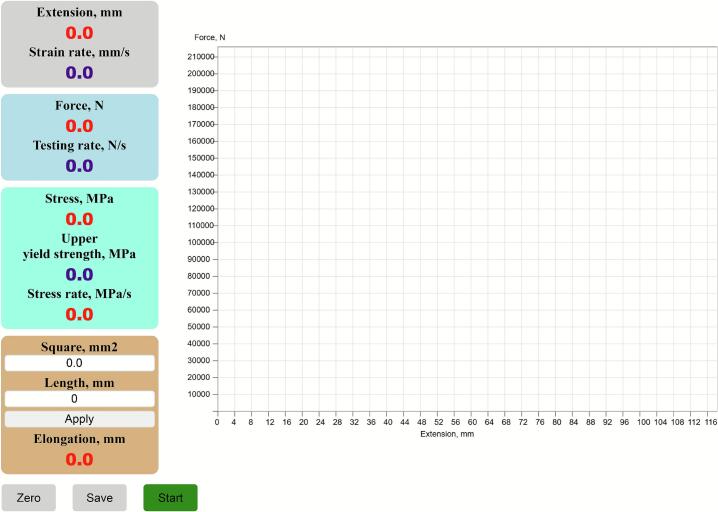
Graphical User Interface.

In the web browser, the user enters the URL or IP address of the web server. A request is sent to the communication module’s web server. In response, an HTML page with embedded JavaScript functions is loaded. This page contains the graphical user interface (GUI) (Fig. 8).

Once the GUI is loaded, the user enters the specimen’s geometrical parameters. These include its diameter and initial gauge length, which are input into the corresponding text fields.

To start data acquisition, the user presses the “Start” button. The button automatically changes to “Stop.” At this point, a connection is established with the communication module via the WebSocket protocol.

**Fig. 9:**
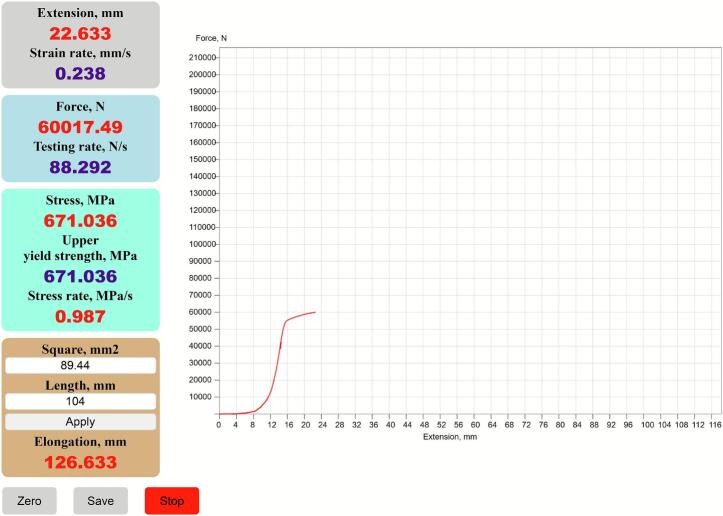
Graphical User Interface during testing.

Upon successful connection, data on specimen extension and applied force begin to stream to the personal device. The acquired raw data and derived values are displayed numerically in text fields and graphically as a force–extension curve (Fig. 9).

To set a reference zero, the user presses the “Zero” button. At this moment, the internal data array is cleared, and the graphical area is reset. Further calculations of extension and force are then performed as differences between new data and the values recorded at the zeroing point.

**Fig. 10:**
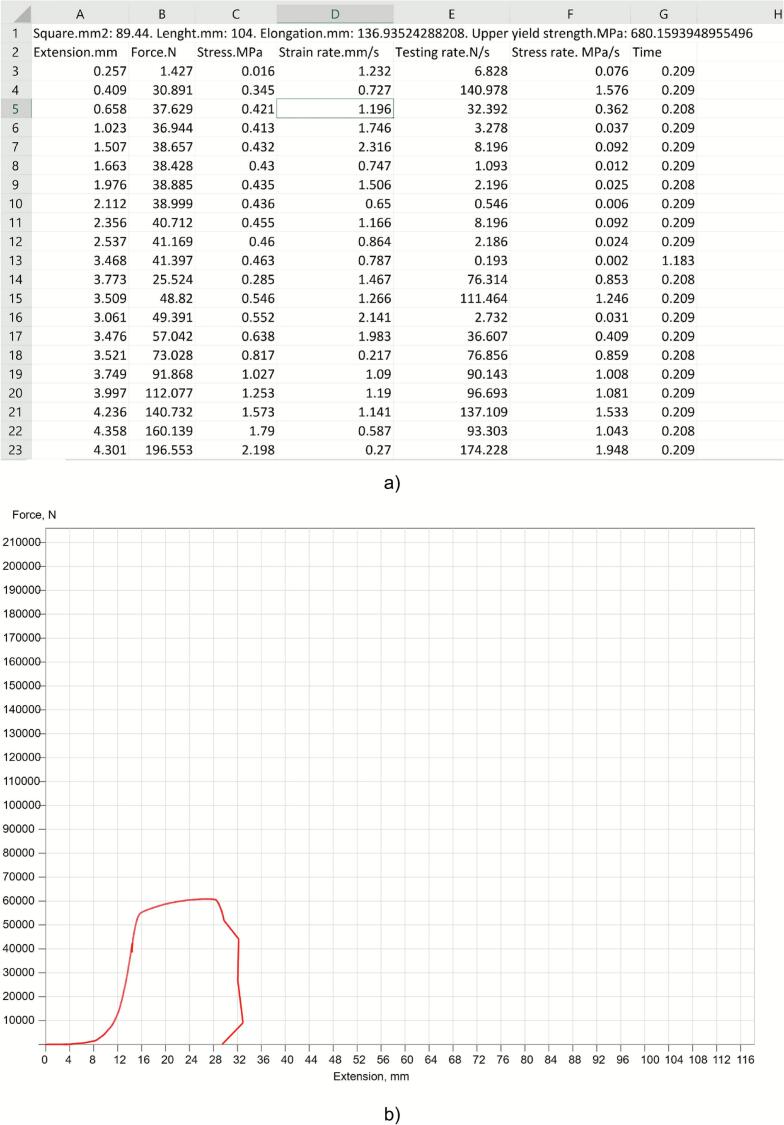
Screenshots of saved data in respective formats: (a) text format (CSV); (b) graphical image (PNG).

After completing the experiment, the user presses the “Stop” button. This action terminates the connection with the communication module. The accumulated numerical and graphical data can then be saved on the user’s personal device. To do this, the user presses the “Save” button. The experimental results are stored in text format and as graphical images (Fig. 10a, 10b).

To start a new experiment, the user presses the “Start” button again. The use of the web platform is generally safe. It does not require specialized equipment or software on the client side. However, certain limitations exist. An excessive number of simultaneous connections may overload the communication module. This can cause delays in data transmission. Prolonged experiments may also lead to increased memory usage on personal devices, and these factors should be considered when organizing collaborative research.

## 6 Validation and characterization

To verify the functionality of the developed web platform, a series of tensile strength tests of steel reinforcement bars was conducted. During these tests, 15 users were simultaneously connected to the communication module.

The experiments followed the recommendations of standard [13]. This standard prescribes strain rate values within the range of 6–60 MPa/s. For this strain rate range and the specified number of simultaneous users, the optimal sampling and transmission frequency was determined to be 15 Hz. This frequency enables uninterrupted and lossless data acquisition, while ensuring that the processor and RAM load of the Raspberry Pi 5 remains below 30 %.

**Fig. 11:**
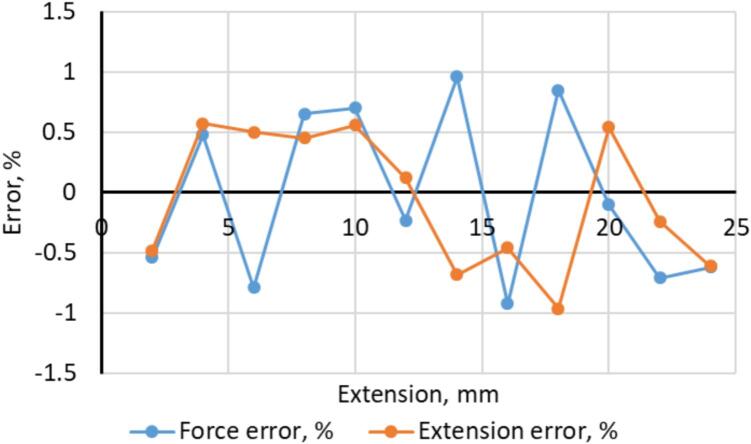
Measurement error of force and extension as a function of extension.

The operability and accuracy of the developed web platform were validated by conducting parallel tests with a standard tensile testing machine. The obtained “force–extension” diagrams from both systems were compared. The comparison showed that deviations in the measured parameters did not exceed the permissible limits for laboratory experiments. The error graphs for force and extension as a function of absolute force (Fig. 11) demonstrate that the error remained stable. It did not exceed ± 1 % for force and ± 1 % for extension. These results meet the requirements of accuracy class 1 of ISO 6892–1 [13].

In addition to the quantitative error evaluation presented in Fig. 11, several sources of measurement uncertainty inherent to the hardware architecture were analyzed. The primary contributor is the nonlinearity of the potentiometers used for extension and force sensing. Although their nominal linearity error does not exceed 0.5 %, small deviations in the slope of the transfer characteristic introduce systematic offsets. These offsets are especially noticeable at larger displacements.

Quantization noise of the 24-bit ADS1256 ADC is another factor. However, within the selected gain and sampling configuration (15 Hz), its contribution remained below 0.1 % of full scale. It did not produce visible fluctuations in the output signal.

Mechanical hysteresis in the force transmission system and backlash in the grips also affect repeatability. They contribute to the observed scatter of force error values within the ± 1 % range. These effects become more pronounced at higher forces, consistent with the slight increase in error amplitude at extension levels above 14–18 mm. Despite these sources, the overall uncertainty remained stable across the investigated force range. The combined influence of potentiometer nonlinearity, ADC quantization, and mechanical hysteresis did not lead to cumulative drift. The total error stayed within the limits required for accuracy class 1 according to ISO 6892–1.

**Fig. 12:**
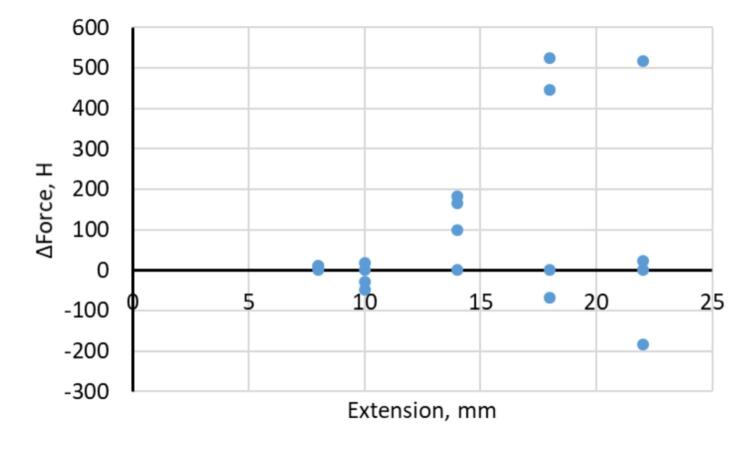
Scatter diagram of force values during repeated measurements.

Additionally, a series of repeated experiments on reinforcement bar specimens was conducted to assess reproducibility. The results of multiple tests demonstrated stable measurements under constant loading conditions. Force fluctuations relative to the reference level remained within limits comparable to standard measuring instruments of accuracy class 1. This is confirmed by the scatter diagram of force values for a given extension level across multiple repetitions (Fig. 12). The diagram illustrates the high repeatability of the system.

Beyond the scatter representation shown in Fig. 12, a quantitative analysis of repeatability was performed using the raw measurement data. For extension levels of 8–10 mm, the force deviations between repeated tests remained within approximately ± 10…±48 N. This corresponds to a relative variation below 0.25 % for the force values typical of this stage of the test.

At higher extensions (14–22 mm), larger deviations were observed (up to ∼ 520 N). This increase is expected due to the rise in absolute force, the nonlinear mechanical behavior of the specimen near yielding, and the higher sensitivity of the pendulum force mechanism to small kinematic irregularities. Even in this range, the deviations remained within 1 % of the typical force values. This fully complies with the accuracy requirements of ISO 6892–1 for class 1 instruments.

Importantly, the distribution of measured force values at each extension level shows no systematic drift between experiments. The values consistently cluster around the same reference level. This indicates stable calibration and the absence of cumulative measurement error. Thus, the observed variability is random in nature and remains within the permissible limits for laboratory measurements. These findings reaffirm the high repeatability of the developed platform.

The obtained results confirm that the developed web platform provides the required level of accuracy and repeatability in accordance with ISO 6892–1. This establishes a solid basis for its further use in laboratory environments.

Web platform specifications:

‒ Data sampling frequency: 15 Hz (67 ms period).
‒ Extension measurement: linear potentiometers with an absolute error not exceeding 10 µm.
‒ Force measurement: relative error within 1 % across a wide operating range.
‒ Data transmission via Wi-Fi wireless network.
‒ Real-time visualization: construction of a “force–extension” curve with extended numerical parameter representation.
‒ Multi-client support (up to 15 users simultaneously).
‒ Data storage: numerical data (CSV) and graphical plots (PNG) for subsequent analysis.
‒ Operation without specialized software — only a standard web browser is required.

Limitations of the web platform:

‒ Exceeding 15 simultaneous connections may cause delays in data transmission.
‒ Long-duration continuous tests (over several hours) require monitoring of memory usage on client devices.

In addition to the listed limitations, several practical factors related to wireless communication should be considered. Laboratory environments often contain numerous metal structures, electrical equipment, and overlapping Wi-Fi networks. These factors may introduce radio-frequency interference, partial signal shielding, temporary packet loss, or reduced throughput under crowded network conditions.

In our experiments, the platform operated in the 2.4 GHz band within a laboratory containing significant metal infrastructure. Nevertheless, due to the short distances between client devices and the communication module, and the limited number of simultaneous connections (up to 15), the wireless link remained sufficiently stable for real-time data transmission.

The platform also incorporates basic reconnection mechanisms. In the event of a temporary signal dropout, the client-side browser automatically attempts to re-establish the WebSocket connection. Upon reconnection, the server resumes streaming real-time data without requiring the experiment to be restarted. Additionally, during short disconnections, measurement data continue to be collected locally on the Raspberry Pi. This minimizes the risk of data loss.

Future improvements may include optional Ethernet fallback, adaptive hotspot mode, or dynamic adjustment of data transmission frequency depending on real-time channel quality.

Thus, the web platform combines high accuracy with ease of use. Its limitations are primarily related to performance and scalability. This makes it an optimal solution for educational and mid-level research tasks. The available performance margin also allows for future expansion of the platform’s functionality.

We believe that our development will make experimental investigations of material strength more accessible and convenient. It can serve as a valuable tool for students conducting laboratory work.

The proposed multi-client web platform, based on Raspberry Pi 5 and the ADS1256 ADC, provides real-time acquisition and transmission of material mechanical property data with visualization in a web browser. No specialized software is required on the client side. Tensile tests on steel reinforcement bars confirmed the platform’s stability and its capability to support simultaneous multi-user access. The platform is a flexible and accessible tool suitable for both scientific research and educational tasks. It can be adapted for various types of materials and sensor systems. Further customization allows investigations of composite materials, polymers, or other materials in educational and research laboratories.

## Ethics statements

We confirmed that our work does not involve any animal or human experiments.

## CRediT authorship contribution statement

**Oleg Ivanov:** Software, Methodology, Writing – review & editing, Supervision. **Volodymyr Shulgin:** Supervision, Methodology, Conceptualization. **Nataliia Popovych:** Visualization, Investigation. **Liudmyla Bondar:** Visualization, Methodology.

## Declaration of competing interest

The authors declare that they have no known competing financial interests or personal relationships that could have appeared to influence the work reported in this paper.
